# Job Satisfaction and Perceived Health in Spanish Construction Workers during the Economic Crisis

**DOI:** 10.3390/ijerph15102188

**Published:** 2018-10-07

**Authors:** Yolanda Navarro-Abal, Luis Carlos Sáenz-de la Torre, Juan Gómez-Salgado, José Antonio Climent-Rodríguez

**Affiliations:** 1 School of Labour Sciences, University of Huelva, Avda, 3 de marzo s/n, 21007 Huelva, Spain; yolanda.navarro@dpsi.uhu.es (Y.N.-A.); luis.saenz@dpces.uhu.es (L.C.S.-d.l.T.); jose.climent@dpsi.uhu.es (J.A.C.-R.); 2 School of Nursing, University of Huelva, Avda. 3 de marzo s/n, 21007 Huelva, Spain; 3 Safety and Health Posgrade Program, Universidad Espíritu Santo, Samborondón (Guayaquil) 091650, Ecuador

**Keywords:** job satisfaction, occupational health, construction industry, work-life balance, work performance, psychosocial factors, occupational risks, occupational risk prevention

## Abstract

The attitude towards work, either satisfaction or dissatisfaction, could influence the way in which workers perceive their health status. To check this hypothesis, this study analyses job satisfaction and its relationship with occupational health perception of Spanish construction workers. A descriptive, cross-sectional, observational study was carried out through a socio-demographic data questionnaire, the General Scale of Job Satisfaction and the SF-36 Health Survey. The study was conducted from January 2014 to June 2015, on a sample of 302 individuals belonging to Andalusian companies using the stratified random method to access companies from different provinces and sizes. The findings indicate that work experience in the sector increases general satisfaction: workers above 55 years of age are more satisfied with their work than those between 36 and 45. Likewise, workers with an experience of 2–5 years show higher levels of overall satisfaction, in opposition to workers with 6 months and 2 years of experience. On the other hand, workers without a contract and interns are the most dissatisfied in job terms. Also, there is a positive correlation between job satisfaction and the positive dimensions of health perception (physical functioning and physical and social role functioning), as well as a negative correlation between job satisfaction and bodily pain and general health perceived.

## 1. Introduction

Employees’ satisfaction has been the subject of multiple investigations since the second half of the 20th century [1]. This is mainly due to the consequences it entails for organisations and due to its impact on performance, absenteeism, and occupational health. A satisfied employee develops behaviours towards a greater commitment and loyalty to the company [2].

The term “job satisfaction” is related to the concepts of quality of life and occupational health [3]. Job satisfaction, given its influence on the employees’ attitudes towards work, is one of the main working life quality indicators, and the foundation of its existence lies in the work’ capacity to meet certain workers’ needs, not only from an operational approach, but also from a wider perspective, considering the social, personal, economic, and hygienic factors. Some authors such as Davis and Newstrom [4] consider satisfaction as a multidimensional phenomenon, while Schermerhorn, Hunt and Osborn [5] considered satisfaction as the degree of the workers’ feelings towards their work.

For Gibson et al. [6], there are five factors causing satisfaction: pay, job, promotion opportunities, supervision, and co-workers. In addition, Zohar [7] considered safety climate as a way of measuring the degree to which safety is perceived by employees and as an important factor in job satisfaction.

The construction sector is characterised by frequent accidents, generally not very serious, from multiple danger sources [8]. When, in the company, a positive safety climate is perceived, the worker understands that a basic need is met, i.e., having safe working conditions leads to positive feelings towards the work [9,10]. Safety and social needs are the greatest motivators for construction workers, although economic benefits appear to remain the greatest source of satisfaction and dissatisfaction. However, more recent studies highlight social needs as the most decisive factors [11]. Other authors have determined the importance of a work-life balance in relation to job satisfaction, task performance and safety climate [12]. More recent studies suggest even transformational leadership as the most suitable model for generating safety climates and satisfaction in the construction industry [13].

It seems that the most qualified construction workers show a greater desire for intrinsic rewards (satisfaction for completed work, challenging tasks, and sense of achievement), while unskilled workers prefer extrinsic rewards (money, position safety, and hygiene safety). With respect to qualified professions’ workers, they show a preference for the combination of intrinsic and extrinsic rewards [14].

Uwakweh [15], in a study on American construction workers, concludes that job satisfaction is conditioned by the balance between what workers expect to get and what they really end up getting in their job. The most important perceived factors are those related to the nature of the work itself, for example, the intrinsic rewards obtained: performing the job in a traditional way, carrying out motivating work, the performance levels executed, and the received feedback.

Baldry [16] notes that many workers in Great Britain feel better about themselves after a productive work day where they can demonstrate their abilities, especially when these are appreciated by others. Regarding pay, they express the need for it to be regularized and stabilized, as well as a higher wage differentials application on the basis of the workers’ qualifications, thus reducing economic incentives based on productivity.

In the United States, Rowings et al. [17] carried out an important investigation on 4600 construction workers from 30 different professions to get to know their perceptions about their work, careers, and working conditions. As for job satisfaction, they claim that construction workers’ satisfaction levels with their work and economic rewards are relatively low. However, a more recent study by Srour et al. [18] obtains different results from those in the study by Rowings et al. [17], where 64% of the 862 interviewed workers claim to be satisfied with their pay and most of them with their professional careers.

Few psychosocial and health studies on construction workers in Spain can be found. The one by López-Araujo y Osca [19] deserves special mention, with 285 workers and an analysis of the functions fulfilled by the control and the social support regarding work stress and some other variables which are indicators of occupational health, taking as a model the Demand-Control-Support model [20,21,22]. The authors of the study highlight that work demands, exposure to harmful conditions, work control and social support are associated with a higher or lower perception of well-being and health at work. They believe that construction companies and organisations should take action to manage work demands, promote workers’ control regarding their job and train supervisory bodies and associates on the importance of social support. A recent study on Chinese construction workers again emphasises the importance of social support regarding the perception of real and perceived occupation health [23].

As reported in the data obtained by the Government of Andalusia [24], the weight of the construction sector in Andalusia's productive structure stood at 5.9% in the year 2014, the lowest since homogeneous data are being reported (1995), and in concordance with the average levels in Spain (5.6%) and in the Euro zone (5.1%). In terms of employment, there has been a shift in the construction sector, from representing 15.3% of Andalusia’s employed workers just before the crisis (2007) to 5.1% in the year of the study (2014). Of the total number of Andalusia’s active workers in the construction sector during the year 2014, 201.200, a 67.14% were employed (135,100 employees), with an unemployment rate of 32.86% in the sector and a total of 66,100 unemployed.

This study implied an analysis of Spanish construction workers’ job satisfaction in the period of the greatest economic crisis the country has ever had since the beginning of its contemporary democratic period (1978), and which has particularly undermined the construction sector, its companies and its workers. Spain’s entry into the European Union meant drastically lowering the monetary interest rates, which led to a real estate bubble that ended with abundant job creation in real estate-associated sectors, construction among them, during the last decades of the 20th century and the first of the 21st century. This high demand for employment in the sector brought along continued substantial improvements in the working conditions, with high salaries and stable recruitment modalities. The crisis of the financial markets of the early 21st century, aggravated in Spain by the bursting of the real estate bubble, ruined much of the improvements achieved in the construction sector in previous years. The situation reached such a virulence during the crisis that in the year 2017, with an evident economic and employment improvement in Spain and the surrounding countries, the level of employment in the construction sector has not yet returned to pre-recession levels, being one of the reasons for the reduction of the employability gap between men and women in Spain after the worst years of precariousness, being the construction sector a particularly masculine one [25].

Specifically, this study has put special emphasis on the workers’ job satisfaction and their occupational health perception relationship. Individuals’ self-assessment of their health status is considered one of the best overall health indicators, as it encompasses the different health dimensions (physical, emotional, social, etc.) [26]. Studies such as the one by Bustos et al. [27] already showed a relationship between the different psychosocial risk factors in Chilean workers’ self-perception of health.

Thus, it is possible to estimate that, as authors have previously indicated [28], relationships between the two variables can be established, so that the attitude, beliefs and values shown by workers in their job, in the form of satisfaction and dissatisfaction, will necessarily influence their behaviours, results and the way in which they perceive their health status.

## 2. Materials and Methods

### 2.1. Design

A descriptive, cross-sectional, observational study was conducted through a sociodemographic information questionnaire, the General Job Satisfaction Scale by Warr, Cook and Wall [29], and the health scales of the SF-36 Health Survey. The study was carried out from January 2014 to June 2015.

### 2.2. Sample

According to the Spanish Statistics Institute’s population survey, the population occupied in construction stood between 135,100 and 150,300 people in Andalusia on average in the years 2014–2015, which meant 15% of the total in the sector in Spain [24]. The sample of construction workers was obtained in Seville and Huelva, the two most western provinces of the Regional Government of Andalusia, where the total of active population who worked in construction was: Seville (30,700–32,700 workers) and Huelva (8900–9800 workers) [30]. A sample of 392 workers was obtained, excluding 90 of them for inadequately completing the questionnaire, thus leaving a total of 302 subjects: 180 from the province of Huelva and 123 from Seville. 83.50% were men (*n* = 255 men), compared to 16.50% of women (*n* = 47). The sample’s calculation was estimated for a proportion, and a convenience selection was used. The access to the sample was made through the contacts established by the research team with the Ministry of Employment of the Government of Andalusia, the competent administration in the area of Occupational Risk Prevention in Andalusia. Through this public administration, it was possible to obtain the construction sector companies agreement of these two provinces to collect the data of those workers who agreed to complete the study questionnaires.

This study was submitted to the Ethics Committee of this public institution and has the ethical approval and certification issued by the competent labour administration of Andalusia.

### 2.3. Procedure

The selection of construction companies was carried out using the stratified random method in order to access companies from different points of the two studied provinces and different size (small, medium and large companies). For the selection of companies, the geographical location was used as criterion, distinguishing Huelva and Seville companies, both from the capital and the rest of the province, according to the greater accessibility possibilities. There were also active construction workers who participated in training programs for the Construction Labour Foundation and one of the most representative unions in the sector in Spain (General Workers' Union). Both in the sampled companies and among the subjects participating in the training programs, the objective of this research was sufficiently explained, guaranteeing confidentiality and its voluntary and anonymous character in the whole procedure, both of the company’s data and the workers’. The data began to be collected initially from January 2014 to December 2014. Given the heterogeneity of the sample and the encountered difficulties for the proper management of the tests, to ensure a greater number of participants and results validity it was decided to continue with data collection until June 2015. At this time, due to an economic improvement that started taking place in this country, that was considered to possibly skew the interest and objective of this work, it was decided to stop the data collection. 

To collect the workers’ data, their company sent them a notification, in paper, and via electronic mail and mobile messaging, explaining the objective of the study and emphasising the voluntary and anonymity character of the same. In addition, anyone who wanted to participate was requested to inform the Human Resources department of his/her company. Once the lists of workers who wanted to participate in the study were obtained from each company, they were individually cited, in a specially appointed room for the study, by a team researcher, and the data collection questionnaire was administered.

### 2.4. Instruments

- Socio-demographic variables. Data collection protocol in which age, sex, working experience time and types of contract are collected.
- General Job Satisfaction Scale by Warr, Cook and Wall [29], translated to Spanish and adapted by Pérez-Bilbao and Fidalgo [31]. It measures job satisfaction through a set of intrinsic and extrinsic factors that reflect the perception of the worker of a paid employment through a series of affective responses about the content of the work itself. It consists of two scales that measure both intrinsic factors, formed by seven items (numbers 2, 4, 6, 8, 10, 12 and 14), and extrinsic factors, which constitute eight items (numbers 1, 3, 5, 7, 9, 11, 13 and 15). The answer format is a 7-point Likert-type scale.
- Health Scales of the SF-36 Health Survey, translated to Spanish and adapted by Alonso, Regidor et al. [32]. Composed of 36 instrument items, these are distributed through 8 scales representing the following health concepts: physical functioning, physical role functioning, bodily pain, general health perceptions, vitality, social role functioning, emotional role functioning and mental health, in addition to a question whose answer is not estimated for the calculation of results, but which does provide useful information about the perceived change in health status during the year prior to the administration of the SF-36 Health Survey. The responses are encoded by scores on a scale ranging from 0 to 100, with “0” being the worst health status for that dimension and “100” the best.

### 2.5. Statistics

Firstly, a variance analysis between the variables objects of study is carried out, that is, job satisfaction and the variables age, type of contract, work experience and work day. Likewise, a Pearsons’ correlation matrix has been carried out between the job satisfaction and the health perception variables.

## 3. Results

Now, the results will be presented together according to each main dimension of interest.

### 3.1. Job Satisfaction and Age

The results of the relationship between satisfaction and age are displayed in Table 1. Once the Levene’s test was carried out, the average equality hypothesis (*p* > 0.05) was accepted. Therefore, there were no significant differences in the job satisfaction perception according to the age of the subjects. However, taking into account descriptive data, the results indicate that the highest levels of overall satisfaction (*M* = 69.33, *SD* = 25.10) and intrinsic satisfaction (*M* = 32.06, *SD* = 10.71) are observed in subjects over 55 years of age. Likewise, it is the subjects between 36 and 45 years who obtain lower levels of general satisfaction (*M* = 61.47, *SD* = 20.55), intrinsic satisfaction (*M* = 28.94, *SD* = 10.04), and extrinsic satisfaction (*M* = 32.42, *SD* = 10.59).

### 3.2. Job Satisfaction and Type of Contract

In relation to job satisfaction and its relationship with the type of contract, the Levene’s test indicates that in the three dimensions of job satisfaction, the hypothesis of the equality of means (*p* > 0.05) must be rejected, i.e., statistically significant differences are identified in the three study variables (general, extrinsic, and intrinsic satisfaction) (Table 2).

The post-hoc comparisons indicate that, in relation to general satisfaction, the most significant differences are found among the subjects working without a contract (*M* = 41.636, *SD* = 19.438) and those who are permanent workers (*M* = 65.566, *SD* = 21.001), permanent seasonal workers (*M* = 67.290, *SD* = 27.319), temporary with a training contract (*M* = 67.096, *SD* = 19.232) and temporary workers (*M* = 67.021, *SD* = 18.384). However, although there are no significant differences with the rest of the labour links, there is a greater dissatisfaction in people without a contract compared to civil servants (*M* = 59.000, *SD* = 2.828) and interim staff (*M* = 68,285, *SD* = 29.233). Only interns show more dissatisfaction than workers without a contract (*M* = 37.500, *SD* = 3.535). As for intrinsic satisfaction, the significant differences found are among workers without a contract (*M* = 20.750, *SD* = 9.863) and permanent seasonal workers (*M* = 32.878, *SD* = 13.806), temporary workers with a training contract (*M* = 31.709, *SD* = 8.959) and temporary workers (*M* = 31.167, *SD* = 9.448). However, although the findings indicate that there are no significant differences, the rest of the contracts, except the interns’ ones (*M* = 18,000, *SD* = 0.002), present greater intrinsic satisfaction than workers without a contract, that is, permanent (*M* = 31.030, *SD* = 10.014), civil servants (*M* = 28.000, *SD* = 0.000), and interim staff (*M* = 30.000, *SD* = 15.220).

Finally, regarding extrinsic satisfaction, the significant differences have been found between working without a contract (*M* = 21.909, *SD* = 9.863) and being a permanent worker (*M* = 34.533, *SD* = 11.174), permanent seasonal worker (*M* = 34.709, *SD* = 13.547), temporary with a training contract (*M* = 35.735, *SD* = 10.782), temporary worker (*M* = 35.027, *SD* = 9.691) and interim workers (*M* = 38.285, *SD* = 14.126). Similarly, as in the previous dimensions, although there are no statistically significant differences, people who work without a contract present a lower extrinsic satisfaction than that of the civil servants (*M* = 31.000, *SD* = 2.828), following the fact that interns again show a lower extrinsic satisfaction level (*M* = 19.500, *SD* = 3.535) (Table 3).

### 3.3. Job Satisfaction and Work Experience

In relation to the work experience, the Levene’s test reports that the hypothesis of equality of means is rejected, not finding differences in relation to work satisfaction and this variable. Nevertheless, it is appreciated that it is the group with a 2 to 5 years of work experience that shows higher levels of general satisfaction (*M* = 70.40, *SD* = 18.06), extrinsic (*M* = 33.05, *SD* = 8.37) and intrinsic satisfaction (*M* = 37.09, *SD* = 10.23). In the same way, the results indicate that subjects with less general satisfaction (*M* = 60.26, *SD* = 21.87), intrinsic (*M* = 27.29, *SD* = 12.78) and extrinsic satisfaction (*M* = 31.33, *SD* = 10.29) are those who have a work experience between 6 months and 2 years (Table 4).

### 3.4. Job Satisfaction and Working Day

In terms of job satisfaction and working day, the Levene’s test indicates that the hypothesis of equality of variances is accepted, with no significant differences for any of the variables. Nevertheless, it is the group of subjects granted with half-time working hours that shows a higher level of general satisfaction (*M* = 73.00, *SD* = 23.17) and of extrinsic satisfaction (*M* = 38.57, *SD* = 13.62). Likewise, the highest levels of intrinsic satisfaction are shown in subjects granted with full-time working hours’ reduction (*M* = 32.76, *SD* = 10.68). At the same time, the lowest levels of general satisfaction (*M* = 63.50, *SD* = 25.68) and intrinsic satisfaction (*M* = 30.00, *SD* = 12.44) are observed in subjects granted half-time working hours. In the same way, the lowest levels of extrinsic satisfaction are found in subjects granted full-time working hours (*M* = 34.15, *SD* = 10.54) (Table 5).

### 3.5. Job Satisfaction and Occupational Health Perception

Now, the results obtained in relation to job satisfaction and the perception of health are exposed, observing positive correlations between the job satisfaction variable and the dimensions: physical functioning (*r* = 0.255 (*), physical role functioning (*r* = 0.141 *) and social role functioning (*r* = 0.199 **), as well as negative correlations with bodily pain (*r* = −0.398 **), and general health perceptions (*r* = −0.178 **). In relation to intrinsic satisfaction, positive correlations are observed with physical functioning (*r* = 0.219 **) and social role functioning (*r* = 0.191 **), and negative ones with bodily pain (*r* = −0.381 **) and general health perception (*r* = −0.171 **). In terms of extrinsic satisfaction, positive correlations with physical functioning (*r* = 0.247 **), physical role functioning (*r* = 0.153 *) and social role functioning (*r* = 0.195 **) are perceived, and negative correlations with bodily pain (*r* = −0.382 **) and general health perception (*r* = −0.171 **) (Table 6).

## 4. Discussion

First, and before stating our reflection on the most specific results of this study, we would like to highlight the advantages that a stratified selection method offers. It is worth mentioning the capacity of a greater inference within a stratum and the comparisons between these strata. On the other hand, it presents smaller random selection errors. Likewise, it also obtains a more representative sample and assures that all the elements of each stratum are represented in the sample. All these advantages have led to a deeper knowledge of the study population.

With this study, it is proven how, in a context of generalized economic crisis and within a sector so influential in the country as the construction, the workers’ sociodemographic, working conditions and psychosocial characteristics influence the perception they have of their working conditions, health and emotional well-being, and therefore, their job satisfaction.

Although no significant differences have been found regarding the job satisfaction level in relation to this study’s Spanish construction workers age, descriptive differences that deserve special consideration have been found. In particular, it is the most veteran workers, with over 55 years of age, who show greater levels of overall satisfaction and that particularly feel more satisfied with those rewards related to intrinsic values of their own work and professional development. These results go in line with other works [33], that also found that age is not a particularly discriminatory variable in relation to job satisfaction in banking employees, although in that study the authors observed a slight increase in the workers’ level of job satisfaction as they were younger, unlike what was found in the present study. However, other studies on different groups of workers show a very similar behaviour to that found in the one in hand with construction workers, indicating a certain tendency to consider that as age increases, workers feel more satisfied and motivated to get involved in the work they develop, valuing the internal factors of the employment relationship to a greater extent [34,35] and reaching maximum satisfaction in the interval prior to retirement [36]. On the other hand, there are authors who emphasise that this satisfaction will depend on the positive or negative attitude shown by the worker towards his/her impending retirement [37].

In relation to the results found that suggest a certain dissatisfaction in workers from 36 to 45 years of age, this could be explained as a consequence of the economic crisis context, and the particularly undermined construction sector. In this sense, studies such as those carried out by Bugard, Brand and House [38] confirm that job insecurity and fear of losing their jobs due to reasons beyond the workers (in the case of our study, this fear was clearly caused by the serious crisis situation that permeates the construction sector in Spain) cause labour dissatisfaction and effects on the workers’ perceived health. From this, we understand that this situation will particularly affect workers that, because of their age, will have difficulty in managing professional recycling and regaining employment once left unemployed, even though they still have and active working life ahead. This is even more so in a context of crisis and massive job destruction in the construction sector.

As for the type of contract to which the construction worker is linked, the study notes that those who worked without a contract, in general, were more dissatisfied at work than the rest of the workers who did have a contract, indifferent of the more temporary or stable type. The only exception found is in interns, who show an even greater job dissatisfaction than the workers without a contract. Other studies that relate job satisfaction with contracts typologies and which also incorporate the category of interns describe very similar results to those found in the present study [39], and this can probably be explained taking into account the pre-labour-market condition of interns, their insufficient regulation in the Spanish legislation, the little social recognition of this figure and the frequent abuse of interns’ working conditions.

In relation to extrinsic and intrinsic job satisfaction variables, regarding the type of contract, again the worker without a contract obtains the lowest levels of job satisfaction with respect to the rest of the legally hired ones, with the exception of interns, as it happened with data on general satisfaction. In particular, significant relationships appear, in relation to intrinsic satisfaction, between non-contract workers and permanent, and between temporary and those with training contracts. In the case of extrinsic satisfaction, significant relationships were found between workers without contracts and the other categories of recruitment except that of civil servants. Some studies have investigated the relationship between job satisfaction and the type of contract [40,41,42,43] and from these we know that the more precarious the hiring modality is (being the absence of contract the greatest precariousness possible), the lower the job satisfaction level. But until the date, there seems to be no studies with scientific evidence regarding this relationship in the construction sector.

A study on Spanish workers from several sectors [44] emphasises that levels of job dissatisfaction increase in relation to the lack of a voluntary basis for the employee to establish the typology of contract that best suits him/her: temporary, permanent seasonal or permanent. Thus, if workers are forced to accept this type of contractual relationship against their desire, this will adversely affect their job satisfaction. This same study also relates the results to the fact that, in the Spanish case, temporary contracts lead to a permanent precarious situation in which the worker goes from one temporary contract to another, staying within this contractual arrangement for a long time.

Likewise, in relation to the data obtained from interns, these indicators allow us to ratify the precarious youth employment model that has been set up in Spain [45], as well as in other European countries, with high levels of unemployment. This model of youth employment precariousness, as indicated by Santamaría [46], cannot be exclusively reduced to the period of economic crisis, as many of its characteristics were also present before the economic recession. In the period of economic and employment crises in which the study was developed, both structural factors (that make precariousness a central element of the labour market) and institutional ones (formed by the distribution of public resources in a welfare state) have an influence through public employment policies. In this way, the results of this work confirm how having a contract (indifferent of its type) and not having it is a clear factor of certain job stability or of total instability, and above all, an element that determines the worker’s level of satisfaction or dissatisfaction.

There are no significant differences in the job satisfaction level expressed by the study subjects in relation to their greater or lesser work experience in the construction sector. However, it is found, at a descriptive level, that the group with work experience between 2 and 5 years shows the highest levels of general satisfaction, both extrinsic and intrinsic. In the same way, the results describe that the subjects with less general satisfaction, intrinsic and extrinsic, is the group formed by those who have a work experience between 6 months and 2 years. The results found are in the line with other already classic studies that relate job satisfaction and professional experience [47,48]. Although other authors do find some indications of greater dissatisfaction as the work experience in the position decreases [49], which could be related to the hypothesis of Rodríguez and Prieto [50], when they point out that the most inexperienced workers are those who have worse working conditions and therefore generate higher levels of dissatisfaction. Another explanation is the one indicated by Bedoya et al. [51] in a work carried out on a teaching group. According to these authors, an increase of the workers’ experience and seniority can lead to an increase of job commitment and satisfaction. The explanation is determined by a feeling of belonging on the part of the professionals that is, in turn, a consequence of a greater perception of self-efficacy in the roles played-out and a greater participation in the organisation’s decisions.

Other authors such as Navarro, Roe and Artiles [52] analyse the concept of temporal focus from a broader perspective. They study its influence on the work experience and job satisfaction, and the important effects it has on the behaviours in the workplace.

The data indicate that there are no statistically significant differences in relation to the working day. However, it is the group of subjects granted half-time working hours’ reduction that show a higher level of general satisfaction and extrinsic satisfaction. Likewise, the highest levels of intrinsic satisfaction are shown in subjects granted full-time working hours’ reduction. In parallel, the lower levels of general and extrinsic satisfaction are obtained by half-time granted workers, and workers granted full-time working hours show lower levels of intrinsic satisfaction. The scientific literature agrees to accept the existence of positive and meaningful relations between work-life balance (through rationalisation measures of times and working hours) and job satisfaction, such as the granted half-time working hours [53,54]. Other studies carried out on the academic collective, with a total of 120 professionals and with the objective of specifically analysing the direct effect of perceived job satisfaction and the work-life balance, showed that job satisfaction tends to be the strongest predictor of academic tasks performance compared to the work-life balance, granting relevance, especially, to the working hours’ reduction measures in order to reconcile personal and family life [12].

In relation to job satisfaction and the perception of health, there is a positive correlation between the job satisfaction variable and the dimensions of physical functioning, physical role functioning and social role functioning, as well as a negative correlation with bodily pain and general health perceived. In other words, the workers in the study show greater job satisfaction as they perceive that carrying out all kinds of physical activities (even the most vigorous) is a greater possibility, without any health limitation (physical functioning). In addition, the workers’ job satisfaction increases as they suffer no physical problems at work or in their daily lives (physical role functioning). Finally, this level of satisfaction also increases as the possibility of carrying out normal social activities does, in and out of their work, without any interference due to physical or emotional problems.

However, the results show that as bodily pain increases and particularly limits the worker, the level of job satisfaction decreases. Similarly, the worse the worker evaluates his/her own health, considering that this will tend to worsen in the future, the lower his/her perception of job satisfaction is [55].

These relations described above are confirmed for the workers’ perception of job satisfaction based on both intrinsic and extrinsic variables, as well as for the general perception of job satisfaction.

In the relationship between job satisfaction and general health perceived, studies such as that by Tejedo [56], with a sample of more than 1000 workers from various activity sectors, found statistically significant differences between job satisfaction and the perception of health. In all of them, a positive perception of health was directly related to high job satisfaction. These results, and therefore those found in the present study, coincide with similar studies in other countries. Thus, Clark [42] finds this same relationship in industrial workers in England, and Lorente, Tordera and Peiró [57] obtain the same results from veteran workers of different European countries. Finally, special emphasis must be put on the relationship between job satisfaction and perception of mental health, where the same results previously described in other studies, such as the one carried out among Mexican professors [58], are found.

The results found in this study can be directly applied by the companies of the construction sector, so that an increase in their workers’ job satisfaction can be made possible, with the proven consequences this will have on the performance, level of commitment, real health and perceived health of their workers, among many other benefits. As has been shown, the implementation of measures aimed at increasing the workers’ level of stability and establishing an adequate system for reconciling working times, that allow them to adequately address their family and social demands, has a clear benefit on their satisfaction. On the other hand, an obvious positive relationship has been established between the perception of good health and job satisfaction. In this sense, the incorporation of healthy programmes in construction companies will undoubtedly cause a positive impact on their workers’ health, who feel healthier and more satisfied in their work.

Precisely, this last line of work could raise promising results after assessing the results on perceived general health and job satisfaction of healthy programmes of construction companies. Although the final sample obtained has been appropriate and it has allowed to obtain relevant, valid and reliable results regarding the studied variables, it is worth highlighting that the original sample on which the study was initially based was larger, so a percentage of workers did not want to participate and, given their eventual participation in the study, these workers could have modified the results in a greater or lesser extent. On the other hand, the fact that this study has been carried out in a period of acute economic recession deserves special mention, being the construction sector a specially affected one, particularly in Spain. These circumstances clearly condition the obtained results, framing them within this specific context. This is why further research is needed on this population but in a period different to the economic recession one.

## 5. Conclusions

Although there are no significant differences regarding age, workers over 55 years show higher levels of overall satisfaction than workers with ages between 36 and 45 years. On the other hand, it is evident that workers without a contract and interns are the most dissatisfied at work. Another idea to emphasise is related to the work experience in the sector, being the group with a work experience between 2 to 5 years the one that shows higher levels of general satisfaction, as opposed to the group of workers with a work experience between 6 months and 2 years. It is also concluded that, although there are no significant differences found, the group of subjects granted a working hours’ reduction on family grounds manifests a higher level of general satisfaction, both extrinsic and intrinsic. At the same time, the lowest levels of job satisfaction are given by the subjects without a working hours’ reduction. Finally, and in relation to the perception of health, there is a positive correlation between the job satisfaction variable and the physical functioning, physical role functioning and social role functioning dimensions, as well as a negative correlation between job satisfaction and bodily pain and general health perceived. These relationships are also applicable to the perception of job satisfaction based on both intrinsic and extrinsic variables.

## Figures and Tables

**Table 1:** Relationship between the general satisfaction dimensions and age.

Job Satisfaction	Levene’s Statistic	df1	df2	Sig.		df	F	Sig.
General Satisfaction	0.63	4	255	0.638	Intergroups	4	1.23	0.296
					Intragroups	255		
					Total	259		
Intrinsic Satisfaction	1.15	4	274	0.332	Intergroups	4	1.14	0.337
					Intragroups	274		
					Total	278		
Extrinsic Satisfaction	0.91	4	264	0.454	Intergroups	4	1.45	0.216
					Intragroups	264		
					Total	268		

**Table 2:** Relationship between the General Satisfaction dimensions and the type of contract.

Job Satisfaction	Levene’s Statistic	df1	df2	Sig.		df	F	Sig.
General Satisfaction	3.24	7	248	0.003	Intergroups	7	2.92	0.006
					Intragroups	248		
					Total	255		
Intrinsic Satisfaction	3.56	7	267	0.001	Intergroups	7	2.39	0.022
					Intragroups	267		
					Total	274		
Extrinsic Satisfaction	2.16	7	257	0.038	Intergroups	7	3.05	0.004
					Intragroups	257		
					Total	264		

**Table 3:** Comparison between groups (post hoc Tukey’s HSD test).

Dependent Variable	(I) What Type of Job Relationship Do You Have Within Your Company?	(J) What Type of Job Relationship Do You Have Within Your Company?	Means Differences (I-J)	Standard Error	Sig.
General Satisfaction	Without a contract	Permanent	−23.93 (*)	7.17	0.022
		Permanent seasonal	−25.65 (*)	7.14	0.009
		Temporary with a contract	−25.46 (*)	7.14	0.010
		Temporary with a training contract	−25.38 (*)	6.37	0.002
		Civil servant	−17.36	15.65	0.954
		Interim	−26.64	9.84	0.126
		Intern	4.13	15.65	1.000
Intrinsic Satisfaction	Without a contract	Permanent	−10.28	3.44	0.061
		Permanent seasonal	−12.12 (*)	3.44	0.012
		Temporary with a contract	−10.95 (*)	3.47	0.038
		Temporary with a training contract	−10.41 (*)	3.06	0.017
		Civil servant	−7.25	7.81	0.983
		Interim	−9.25	4.86	0.551
		Intern	2.75	7.81	1.000
Extrinsic Satisfaction	Without a contract	Permanent	−12.62 (*)	3.74	0.019
		Permanent seasonal	−12.80 (*)	3.72	0.016
		Temporary with a contract	−13.82 (*)	3.68	0.005
		Temporary with a training contract	−13.11 (*)	3.31	0.002
		Civil servant	−9.09	8.16	0.953
		Interim	−16.37 (*)	5.13	0.034
		Intern	2.40	8.16	1.000

**Table 4:** Relationship between the general satisfaction dimensions and work experience.

Job Satisfaction	Levene’s Statistic	df1	df2	Sig.		df	F	Sig.
General Satisfaction	1.578	5	242	0.167	Intergroups	5	0.57	0.723
					Intragroups	242		
					Total	247		
Intrinsic Satisfaction	2.300	5	261	0.045	Intergroups	5	0.79	0.558
					Intragroups	261		
					Total	266		
Extrinsic Satisfaction	1.623	5	250	0.154	Intergroups	5	0.64	0.669
					Intragroups	250		
					Total	255		

**Table 5:** Relationship between the general satisfaction dimensions and working day.

Job Satisfaction	Levene’s Statistic	dg1	df2	Sig.		df	F	Sig.
General Satisfaction	1.761	3	251	0.155	Intergroups	3	0.498	0.684
					Intragroups	251		
					Total	254		
Intrinsic Satisfaction	0.947	3	270	0.419	Intergroups	3	0.477	0.699
					Intragroups	270		
					Total	273		
Extrinsic Satisfaction	2.706	3	259	0.046	Intergroups	3	0.430	0.732
					Intragroups	259		
					Total	262		

**Table 6:** Correlation between job satisfaction and occupational health perception.

Job Satisfaction		PF	PRF	BP	GHP	V	SRF	ERF	MH
General Satisfaction	*r*	0.255 **	0.141 *	−0.398 **	−0.178 **	0.007	0.199 **	0.043	0.057
	*Sig. (bil.)*	0.000	0.024	0.000	0.006	0.907	0.001	0.491	0.373
	*n*	241	256	254	240	253	257	256	249
Intrinsic Satisfaction	*r*	0.219 **	0.103	−0.381 **	−0.171 **	−0.011	0.191 **	0.005	0.011
	*Sig. (bil.)*	0.000	0.087	0.000	0.006	0.858	0.001	0.932	0.857
	*n*	257	274	272	259	271	276	274	266
Extrinsic Satisfaction	*r*	0.247 **	0.153 *	−0.382 **	−0.171 **	0.016	0.195 **	0.042	0.086
	*Sig. (bil.)*	0.000	0.013	0.000	0.007	0.799	0.001	0.491	0.167
	*n*	250	265	263	248	261	266	265	258

Physical Functioning = PF; Physical Role Functioning = PRF; Bodily Pain = BP; General Health Perceptions = GHP; Vitality = V; Social Role Functioning = SRF; Emotional Role Functioning = ERF; Mental Health = MH. * The correlation is significant at 0.05 level (bilateral); ** The correlation is significant at 0.01 level (bilateral).
